# Internet-Based Cognitive Behavioral Therapy and Telephone Coaching for Anxiety in Children

**DOI:** 10.1001/jamanetworkopen.2026.29195

**Published:** 2026-08-14

**Authors:** Andre Sourander, Katri Kaajalaakso, Tarja Korpilahti-Leino, Terja Ristkari, Tiia Ståhlberg, Susanna Hinkka-Yli-Salomäki, Yuko Mori, Terhi Luntamo

**Affiliations:** 1 Research Centre for Child Psychiatry, University of Turku, Turku, Finland; 2 INVEST Research Flagship Centre, University of Turku, Turku, Finland; 3 Department of Child Psychiatry, Turku University Hospital, Turku, Finland; 4 Department of Adolescent Psychiatry, Turku University Hospital, Turku, Finland

## Abstract

**Importance:**

Limited data are available on the long-term effectiveness of internet-based cognitive–behavioral therapy (iCBT) for anxiety among children.

**Objective:**

To evaluate the association of an iCBT intervention with anxiety in children compared with a psychoeducation control group over 12 and 24 months.

**Design, Setting, and Participants:**

This cohort study is based on 2-year follow-up data of the randomized clinical trial Master Your Worries, which was conducted between August 2017 and January 2020. Participants were Finnish school children aged 10 to 13 years identified through population-level screening as exhibiting elevated levels of anxiety. Statistical analyses for this study were performed between June 2, 2025, and June 16, 2026.

**Intervention:**

Participants in the intervention group followed an internet-based therapy program, which comprised 10 modules, including telephone contact with a coach. The control group received psychoeducation in digital form.

**Main Outcomes and Measures:**

The primary outcome was change in participants’ anxiety measured by the total score of the Screen for Child Anxiety Related Disorders–Child and Parent versions (SCARED-C/P). The secondary outcomes were changes in SCARED-C and SCARED-P subscale scores as well as scores on the Child Anxiety Impact Scale, Child Depression Inventory, and Depression Anxiety and Stress Scale–21 Items.

**Results:**

A total of 465 children (median [range] age 11, [10-13] years; 332 [71%] girls) with anxiety symptoms (SCARED total score of ≥22 at baseline) were followed up for 2 years after randomization. On the basis of a linear mixed-effects model for repeated measures, the primary outcomes (SCARED-C and SCARED-P total scores) showed statistically significant differences between the groups at both the 12-month (SCARED-C mean score in the intervention group was 4.5 [95% CI, 2.2-6.8] points higher; *P* < .001; Cohen *d* = 0.35; and SCARED-P mean score was 3.4 [95% CI, 1.8-5.1] points higher; *P* < .001; Cohen *d* = 0.36) and 24-month (SCARED-C mean score in the intervention group was 4.4 [95% CI, 1.7-7.1] points higher; *P* = .001; Cohen *d* = 0.29; and SCARED-P mean score was 2.3 [95% CI, 0.1-4.5] points higher; *P* = .04; Cohen *d* = 0.19) follow-ups. Overall, the effect size of iCBT increased from 6 months to 12 months, and these results were maintained through 24 months. Statistically significant differences favoring iCBT were also seen in anxiety symptom subgroups. For example, at 12 months, the mean change in social anxiety was 1.6 points higher in the intervention group (95% CI, 1.0-2.3; Cohen *d* = 0.45). No statistically significant differences were seen in parental mental health.

**Conclusions and Relevance:**

In this cohort study of guided iCBT, the outcomes of the intervention extended through 24 months. This suggests a delayed increase in the effectiveness of the iCBT targeting childhood anxiety, which has important implications for future research and clinical practice.

## Key Points

**Question:**

What is the long-term association of internet-based cognitive-behavioral therapy (iCBT) with anxiety among children aged 10 to 13 years?

**Findings:**

In this cohort study of 465 Finnish children, child and parent reports of anxiety showed statistically significant superiority of iCBT compared with psychoeducation at the 12-month and 24-month follow-ups, and the benefits associated with iCBT continued to increase from the 6-month follow-up to the 12-month follow-up.

**Meaning:**

This study suggests that early identification of childhood anxiety combined with iCBT or even digital psychoeducation was associated with significant long-term improvement in children’s symptoms and functioning level for up to 24 months.

## Teaser

This cohort study investigates the the 12- and 24-month outcomes associated with a telephone-guided internet-based cognitive-behavioral therapy intervention compared with digital psychoeducation among Finish children with anxiety.

## Introduction

In the 2020 Practice Guidelines for the Treatment of Anxiety Disorders,^1^ the American Academy of Child and Adolescent Psychiatry highlights the critical shortage of professionals trained in child and adolescent behavioral health. Many children with anxiety disorders do not receive evidence-based treatment due to a shortage of trained professionals, long waiting lists, and concerns related to stigma,^2,3^ barriers that digital interventions could help address. Internet-based cognitive behavioral therapy (iCBT) has demonstrated effectiveness in treating anxiety among children and adolescents.^4^

There are various formats in which iCBT interventions can be delivered, including guided, unguided, or mixed-methods care.^4^ The short-term efficacy of guided iCBT in reducing anxiety symptoms among children has been studied in randomized clinical trials (RCTs),^5,6,7,8,9^ with results demonstrating significantly greater improvement in treatment groups compared with waitlist controls^5,6,8^ or active control conditions, such as internet-delivered child-directed play^7^ or psychoeducation.^9^

However, no previous studies have examined the efficacy of iCBT for childhood anxiety compared with a control group beyond 6 months. In addition, none of the earlier studies were population based. Two Swedish studies^10,11^ reported 12-month outcomes of iCBT for childhood anxiety, but participants in the control condition could cross over to iCBT immediately after the 12-week posttreatment assessment. An study^12^ of adolescents comparing iCBT with traditional CBT, with a 12-month follow-up, found no significant differences in outcome changes between the treatment groups. In these studies,^10,11,12^ participants were either drawn from a clinic-based population or recruited through advertising.

A previous study^9^ reported the 6-month efficacy of iCBT compared with psychoeducation. A total of 234 children with high anxiety levels were identified through a 2-stage population-level screening using a brief 5-item and a full-length 41-item version of the Screen for Child Anxiety Related Disorders–Child version (SCARED-C) questionnaire. Approximately 80% of children who screened at stage 2 met the diagnostic criteria of an anxiety disorder (*International Statistical Classification of Diseases and Related Health Problems, Tenth Revision* [*ICD-10*]) based on the Development and Well-Being Assessment. At 6 months, an analysis comparing the iCBT intervention group and digital psychoeducation control group revealed a statistically significant difference in improvement on the child-reported primary outcome measure (SCARED-C), whereas there were no between-group differences on the Screen for Child Anxiety Related Disorders–Parent version (SCARED-P). Although both groups showed significant improvement from baseline to 6 months, the effect sizes for changes in the primary and secondary outcomes were low when changes in the groups were compared.

The critical limitation in the current research knowledge is the lack of studies evaluating the long-term outcomes of iCBT targeting childhood anxiety. The current study addresses this gap by evaluating the 12- and 24-month outcomes of a telephone-guided iCBT intervention compared with a digital psychoeducation control group. On the basis of the previous studies,^10,11,12^ we hypothesized that the benefits of iCBT would be maintained at least until 24-month follow-up.

## Methods

Detailed information on the study protocol of the original RCT and the Master Your Worries intervention has been previously published.^13^ The original study was registered at ClinicalTrials.gov.^14^ The original study and its follow-up phase were approved by the research ethics board of the Hospital District of Southwest Finland as well as local authorities. Parents provided written informed consent before the health check-up (screening phase). The children provided informed assent during the checkup and were informed about the study by the school nurse. After recruitment and eligibility assessment, written informed consent for the RCT was obtained from the children and their parents via the digital platform. This report follows the Strengthening the Reporting of Observational Studies in Epidemiology (STROBE) reporting guideline for cohort studies.

### Participants

The sample consisted of children aged 10 to 13 years in grades 4 to 6 who were attending comprehensive schools across 6 study sites in Finland. (See eTable 1 in Supplement 1 for demographic characteristics of the intervention and education control groups). Screening for anxiety was conducted as a part of yearly school health checkups between August 2017 and January 2020 using a short 5-item questionnaire based on SCARED-C. Families of children who reported anxiety symptoms were contacted for recruitment. To be in line with the preventive focus of the study, they could be included if the child scored at least 22 on the 41-item SCARED. Additional inclusion and exclusion criteria are presented in eTable 2 in Supplement 1.

### iCBT Intervention and Control Group

In the study, a 10-week iCBT intervention with weekly telephone coaching was delivered to the parents and children in the intervention group (eTable 3 in Supplement 1). Each week, the children and parents were offered separate materials via the digital platform, including texts, images, educational audio clips, animations, and assignments with feedback. As reported previously,^15^ the mean (SD) time spent on the webpage per family per theme was 127 (73) minutes (range, 38-420 minutes). The telephone coaching was highly structured, and the mean (SD) length of the weekly calls was 32 (8) minutes (range, 4-58 minutes).^15^ One month after completing the intervention, families received a booster call, and the digital material remained accessible for up to 2 years from the start of the intervention.

Parents and children in the control group received separate materials via the digital platform, including psychoeducation about anxiety, well-being, healthy life habits, parenting skills, and help-seeking. The material did not include CBT techniques or telephone coaching.

### Screening and Recruitment

**Figure 1.**
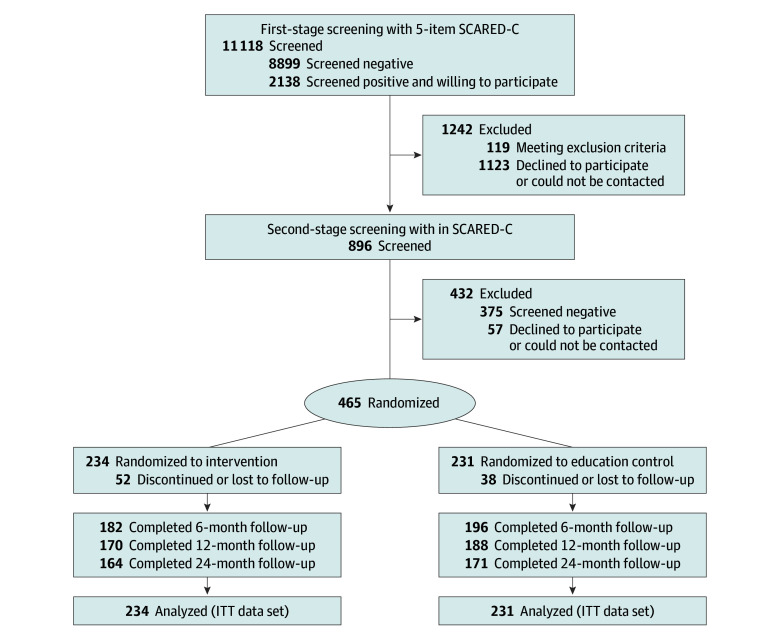
Participant Flow Diagram ITT indicates intention to treat; SCARED-C, Screen for Child Anxiety Related Disorders–Child version.

The flowchart for the study is presented in Figure 1. To ensure the persistence of each child’s anxiety symptoms, we used a 2-stage screening process.^9,13^ Altogether, 11 118 children were screened with a short 5-item version of the SCARED-C (eTable 4 in Supplement 1). Of these children, 2138 (19%) met the screening criteria and were contacted for recruitment. In the second stage of screening, 896 children completed the 41-item SCARED. Of these children, 432 (48%) were excluded due to negative screening results, unwillingness to participate, or loss of contact. A total of 465 children were randomized to 2 groups: 234 in the intervention group and 231 in the control group. The overall response rate in the RCT was 80.6% (n = 375), 77% (n = 358) at the 12-month follow-up and 72% (n = 335) at the 24-month follow-up. To be able to detect a mean difference of 3.0 points between the 2 groups, the sample size of the study was calculated to be 147 children in both treatment groups. To allow for 30% attrition over time, we decided to randomize a minimum of 210 participants to each group.

### Primary Outcome

The primary outcome was the total score of SCARED-C and SCARED-P^16,17^ measuring the child’s anxiety. SCARED includes 41 items divided into 5 subscales: panic disorder, generalized anxiety disorder, separation anxiety disorder, social anxiety disorder, and school phobia. The items are scored on a scale from 0 to 2, with 0 indicating not true or hardly ever true and 2 indicating very true or often true, for a maximum total score of 82. Higher scores indicate higher anxiety. Internal consistency for the Finnish SCARED was α = .92 for the child report and α = .90 for the parent report, and the interrater agreement of child and parent SCARED total scores was 0.37.^18^ A cut-off score of 25 for both children and parents has been determined to indicate a presence of an anxiety disorder.^16^

### Secondary Outcomes and Additional Measures

Secondary outcomes included changes in the SCARED-C and SCARED-P subscale scores; changes in daily functioning assessed with the Child Anxiety Impact Scale-Child (CAIS-C) and Child Anxiety Impact Scale-Parent (CAIS-P) versions (score range, 0-81; higher scores indicate more severe difficulties^19^), child-reported emotional symptoms based on the Child Depression Inventory (CDI; score range, 0-54; higher scores indicate higher levels of depressive symptoms^20^), and parent-reported parental depression, anxiety, and stress symptoms measured with the Depression, Anxiety and Stress Scale–21 Items (DASS-21; score range, 0-63; higher scores indicate more severe psychopathology^21^). To evaluate the presence of any anxiety disorder at baseline, the Development and Well-Being Assessment interview^22^ was conducted via telephone with the child and the parents. Parents also provided information about the child’s use of psychotropic medication and psychosocial support during the 6 months before randomization and at each follow-up because the children were allowed to access treatment as usual. No significant differences were found for diagnostic profiles, mental health service contacts, or psychotropic medication use between the 2 RCT groups. At 12-month follow-up, 61 children (36%) in the intervention group and 85 (44%) in the control group had received psychosocial support (at least 1 visit) or used psychoactive medication. At 24-month follow-up, the respective numbers were 70 (42%) and 79 (46%). There were no statistically significant differences between the groups at 12- or 24-month follow-ups.

Potential sources of bias were addressed by inviting all participants from the original RCT to the follow-up assessment and by comparing baseline characteristics of participants and nonparticipants. Standardized, validated outcome measures were used to reduce measurement bias.

### Statistical Analysis

The primary outcome measures—the total scores from the SCARED-C/P —as well as the secondary variables were analyzed separately with linear mixed models for repeated measurements and reported as model-based least-squares mean differences with corresponding 95% CIs. The models included the treatment group and the stratifying factor (sex) as between-factors and time (6, 12, and 24 months after randomization) as a within-factor. The baseline measurement of each outcome was included as a covariate. The participants were included as random effects to generalize the results beyond our study sample. The restricted maximum likelihood estimation method was applied to account for missing observations in estimating the model parameters. The estimation uses all available data under the assumption of missing at random (MAR). Because MAR is an untestable assumption involving the unobserved outcomes, we conducted ancillary robustness analyses that included modeling without baseline adjustment. To analyze whether missing observations affected the results, a sensitivity analysis was performed using multiple imputation for missing observations. Altogether 25 imputed datasets were generated using the fully conditional specification method under the assumption that data were missing at random. Analyses were performed for each imputed dataset, and the results were combined using the Rubin rules.^23^ Treatment allocation, child’s sex and age, family structure, and parental educational level were included in the imputation model. All randomized children were included in the analyses in accordance with the intention-to-treat principle. There were no baseline differences in the primary and secondary outcome variables. Differences in service use between the intervention and control groups were assessed using the Fisher exact test at both the 12-month and 24-month follow-up assessments. Because the 2 primary outcomes were single measurements from separate informants, adjustment of *P* values due to multiple tests was not necessary. To complement the statistical testing, Cohen *d* values were calculated as a measure of effect size for differences in changes from baseline for the primary and secondary variables between the 2 groups. A 2-sided *P* < .05 was considered statistically significant. PROC MIXED in SAS, version 9.4 (SAS Institute Inc) was used in all statistical analyses.

## Results

**Figure 2.**
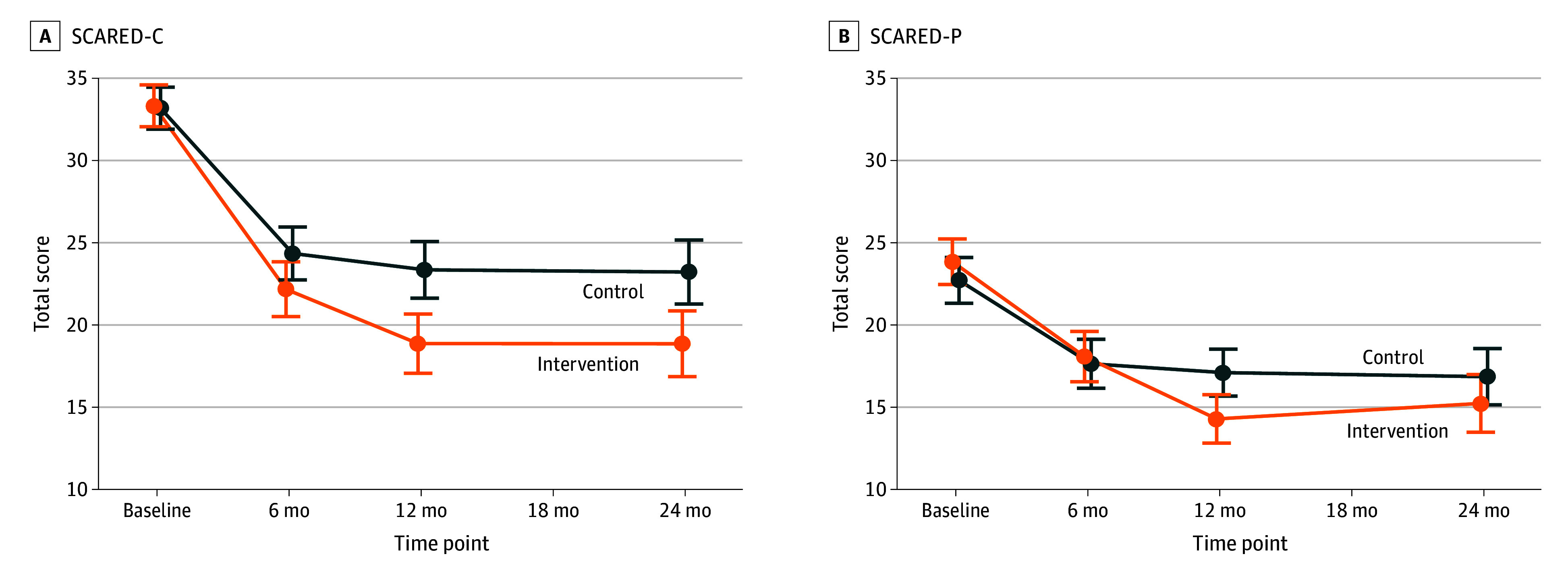
Line Graph of Changes in Screen for Child Anxiety Related Disorders–Child version (SCARED-C) and Screen for Child Anxiety Related Disorders–Parent version (SCARED-P) Scores by Treatment Group Error bars indicate 95% CIs of the least-squares mean.

A total of 465 children (median [range] age, 11 [10-13] years; 332 [71%] girls and 133 [29%] boys) with the SCARED-C total score of 22 or more at baseline were followed up for 2 years after randomization. Of the participating parents, 421 were biological mothers, 39 were biological fathers, and 5 were other caregivers. Both the guided iCBT intervention group and the digital psychoeducational control group showed significant decrease in SCARED-C (mean [SE] change in the intervention group, 14.5 [1.1]; *P* < .001; mean [SE] in the control group, 10.0 [1.1]; *P* < .001) and SCARED-P (mean [SE] change in the intervention group, 8.6 [0.9]; *P* < .001; mean [SE] in the control group, 5.9 [0.9]; *P* < .001) total scores from baseline to 24 months. After the RCT phase, statistically significant decrease in anxiety was seen only for the intervention group from 6 to 12 months (mean [SE] change in SCARED-C score, 3.3 [0.7]; *P* < .001; mean [SE] change in SCARED-P score, 3.8 [0.6]; *P* < .001). The improvements were maintained for the intervention group between 12 and 24 months (SCARED-C mean [SE] change, −0.01 [0.95]; *P* = .99; SCARED-P mean [SE] change, −0.98 [0.69]; *P* = .16) and for the control group from 6 to 24 months (SCARED-C mean [SE] change, 1.1 [0.96]; *P* = .24; SCARED-P mean [SE] change, 0.73 [0.77]; *P* = .34) (Figure 2). Altogether, 130 participants (28%) were unavailable for follow-up.

For the primary outcome, the intervention group showed a mean (SE) decrease of 14.4 (0.9) points (*P* < .001) in SCARED-C total scores from baseline to 12 months and a mean (SE) decrease of 14.5 (1.1) points (*P* < .001) from baseline to 24 months (Table 1). SCARED-P total scores decreased by a mean (SE) of 9.6 (0.7) (*P* < .001) and 8.6 (0.9) (*P* < .001), respectively. In the control group, SCARED-C total scores decreased by a mean (SE) of 9.8 (0.9) (*P* < .001) from baseline to 12 months and by 10.0 (1.1) (*P* < .001) from baseline to 24 months, and SCARED-P total scores decreased by a mean (SE) of 5.6 (0.7) (*P* < .001) and 5.9 (0.9) (*P* < .001), respectively.

**Table 1.** Changes in Primary and Secondary Outcomes From Baseline to 12 and 24 Months After Randomization to the Intervention and Control Groups

Outcome	Intervention				Control			
	Baseline to 12 mo		Baseline to 24 mo		Baseline to 12 mo		Baseline to 24 mo	
	Mean (SE)^a^	*P* value	Mean (SE)^a^	*P* value	Mean (SE)^a^	*P* value	Mean (SE)^a^	*P* value
**Primary**								
SCARED-C total score	14.4 (0.9)	<.001	14.5 (1.1)	<.001	9.8 (0.9)	<.001	10.0 (1.1)	<.001
SCARED-P total score	9.6 (0.7)	<.001	8.6 (0.9)	<.001	5.6 (0.7)	<.001	5.9 (0.9)	<.001
**Secondary: child reported**								
SCARED-C subscale scores								
Panic disorder	2.8 (0.3)	<.001	2.9 (0.4)	<.001	2.3 (0.3)	<.001	2.1 (0.4)	<.001
General anxiety	3.8 (0.3)	<.001	3.2 (0.4)	<.001	2.6 (0.3)	<.001	1.9 (0.3)	<.001
Separation anxiety	4.2 (0.2)	<.001	5.0 (0.2)	<.001	3.1 (0.2)	<.001	4.2 (0.2)	<.001
Social anxiety	2.8 (0.3)	<.001	2.4 (0.3)	<.001	1.2 (0.2)	<.001	1.1 (0.3)	<.001
School phobia	0.9 (0.1)	<.001	1.0 (0.1)	<.001	0.5 (0.1)	<.001	0.7 (0.1)	<.001
CDI total score	2.9 (0.6)	<.001	2.3 (0.7)	.002	2.1 (0.6)	<.001	1.2 (0.7)	.10
CAIS total score	9.8 (0.9)	<.001	8.1 (1.0)	<.001	7.4 (0.9)	<.001	5.4 (1.0)	<.001
**Secondary: parent reported **								
SCARED-P subscale scores								
Panic disorder	1.3 (0.2)	<.001	0.7 (0.3)	.02	0.4 (0.2)	.046	0.4 (0.3)	.18
General anxiety	2.6 (0.2)	<.001	2.5 (0.3)	<.001	1.6 (0.2)	<.001	1.5 (0.3)	<.001
Separation anxiety	3.0 (0.2)	<.001	3.4 (0.2)	<.001	2.1 (0.2)	<.001	2.8 (0.2)	<.001
Social anxiety	1.8 (0.2)	<.001	1.3 (0.3)	<.001	0.8 (0.2)	<.001	0.6 (0.3)	.02
School phobia	0.9 (0.1)	<.001	0.8 (0.1)	<.001	0.5 (0.1)	<.001	0.6 (0.1)	<.001
CAIS total score	7.0 (0.6)	<.001	5.3 (0.8)	<.001	4.0 (0.6)	<.001	3.6 (0.7)	<.001
DASS-21 scores								
Total	1.8 (0.8)	.03	0.9 (1.0)	.32	1.2 (0.8)	.13	0.7 (0.9)	.44
Depression	0.4 (0.4)	.23	−0.1 (0.4)	.82	0.6 (0.3)	.09	0.3 (0.4)	.49
Anxiety	0.6 (0.3)	.03	0.6 (0.3)	.047	0.3 (0.3)	.29	−0.02 (0.3)	.96
Stress	0.7 (0.4)	.07	0.4 (0.4)	.34	0.4 (0.4)	.34	0.5 (0.4)	.21

Abbreviations: CAIS, Child Anxiety Impact Scale; CDI, Child Depression Inventory; DASS-21, Depression Anxiety and Stress Scale–21 Items; SCARED-C, Screen for Child Anxiety Related Disorders–Child version; SCARED-P, Screen for Child Anxiety Related Disorders–Parent version.

^a^ Least-squares mean.

For secondary outcomes, both groups had significant changes in anxiety level from baseline to 12 months and between baseline and 24 months in almost all child- and parent-reported secondary outcome measures, including in anxiety subscales of SCARED-C and SCARED-P, daily functioning (CAIS-C and CASIS-P), and child-reported depression (CDI) (eTable 5 in Supplement 1). Parent self-reports of mental health problems showed significant decrease in the intervention group between baseline and 12 months in DASS-21 total score (mean [SE] change, 1.8 [0.8]; *P* = .03) and anxiety score (mean [SE] change, 0.6 [0.3]; *P* = .03) of which the change in anxiety scores remained significant at 24 months (mean [SE] change, 0.6 [0.3]; *P* = .047).

Table 2 presents between-group comparisons of changes in primary and secondary outcome measures between baseline and 12 months and baseline and 24 months. The intervention group showed a significantly larger change in both primary outcomes. At 12 months, the mean change in the SCARED-C total score was 4.5 (95% CI, 2.2-6.8) points higher (*P* < .001; Cohen *d* = 0.35) in the intervention group than in the control group, and the mean change in the SCARED-P total score was 3.4 (95% CI, 1.8-5.1) points higher (*P* < .001; Cohen *d* = 0.36). Between baseline and the 24-month follow-up, the mean change in the SCARED-C total score was 4.4 (95% CI, 1.7-7.1) points higher (*P* = .001; Cohen *d* = 0.29) in the intervention group vs control group, and the mean change in SCARED-P score was 2.3 (95% CI, 0.1-4.5) points higher (*P* = .04; Cohen *d* = 0.19). As an ancillary robustness analysis, eTable 6 in Supplement 1 shows the same comparisons using analysis of variance. These results support those of the primary analysis.

**Table 2.** Between-Group Changes in Primary and Secondary Outcomes at 12 and 24 Months^a^

Outcome	Intervention vs control					
	12-mo Follow-up			24-mo Follow-up		
	Mean (95% CI)^b^	*P* value	Cohen *d*	Mean (95% CI)^b^	*P* value	Cohen *d*
**Primary **						
SCARED-C total score	4.5 (2.2 to 6.8)	<.001	0.35	4.4 (1.7 to 7.1)	.001	0.29
SCARED-P total score	3.4 (1.8 to 5.1)	<.001	0.36	2.3 (0.1 to 4.5)	.04	0.19
**Secondary: child reported**						
SCARED-C subscale cores						
Panic disorder	0.4 (−0.4 to 1.2)	.35	0.08	0.8 (−0.2 to 1.8)	.11	0.14
General anxiety	1.0 (0.2 to 1.7)	.01	0.23	1.1 (0.2 to 2.0)	.02	0.22
Separation anxiety	1.2 (0.7 to 1.7)	<.001	0.41	1.0 (0.5 to 1.5)	<.001	0.34
Social anxiety	1.6 (1.0 to 2.3)	<.001	0.45	1.4 (0.7 to 2.1)	<.001	0.34
School phobia	0.3 (0.01 to 0.6)	.04	0.18	0.2 (−0.1 to 0.5)	.25	0.10
CDI total score	0.8 (−0.6 to 2.3)	.25	0.10	1.1 (−0.7 to 2.9)	.24	0.11
CAIS total score	3.0 (1.0 to 5.1)	.004	0.26	3.3 (0.8 to 5.7)	.009	0.24
**Secondary: parent reported**						
SCARED-P subscores						
Panic disorder	0.7 (0.1 to 1.3)	.02	0.21	0.1 (−0.6 to 0.9)	.73	0.03
General anxiety	0.7 (0.1 to 1.3)	.02	0.21	0.9 (0.1 to 1.6)	.02	0.21
Separation anxiety	0.8 (0.4 to 1.2)	<.001	0.32	0.5 (0.05 to 0.9)	.03	0.19
Social anxiety	1.0 (0.5 to 1.5)	<.001	0.33	0.7 (0.04 to 1.3)	.04	0.19
School phobia	0.3 (0.02 to 0.5)	.04	0.19	0.1 (−0.2 to 0.4)	.67	0.04
CAIS total score	2.9 (1.5 to 4.4)	<.001	0.35	1.5 (−0.4 to 3.5)	.12	0.14
DASS-21 scores						
Total	−0.1 (−2.1 to 2.0)	.95	0.01	−0.4 (−2.8 to 2.0)	.73	0.03
Depression	−0.3 (−1.2 to 0.6)	.47	0.06	−0.6 (−1.7 to 0.6)	.33	0.09
Anxiety	0.1 (−0.6 to 0.7)	.85	0.02	0.4 (−0.4 to 1.1)	.32	0.09
Stress	0.05 (−1.0 to 1.1)	.93	0.01	−0.4 (−1.5 to 0.7)	.44	0.07

Abbreviations: CAIS, Child Anxiety Impact Scale; CDI, Child Depression Inventory; DASS-21, Depression Anxiety and Stress Scale–21 Items; SCARED-C, Screen for Child Anxiety Related Disorders–Child version; SCARED-P, Screen for Child Anxiety Related Disorders–Parent version.

^a^ Analysis was adjusted for baseline measurement of each outcome as a covariate.

^b^ Baseline adjusted least-squares means.

Table 2 shows the results for secondary outcomes. At 12-month follow-up, there was a higher decrease in anxiety symptoms in the intervention group for all child- and parent-reported anxiety subscale scores, with Cohen *d* = 0.23 for child-reported general anxiety, 0.41 for child-reported separation anxiety, 0.45 for child-reported social anxiety and 0.18 for child-reported school phobia, except for child reports of panic symptoms. At 24 months, child and parent reports of improvements in general, separation, and social anxiety remained significant, with Cohen *d = *0.19 for parent-reported separation anxiety and social anxiety and Cohen *d = *0.34 for child-reported separation anxiety and social anxiety. Changes in CDI scores did not differ between the intervention and control groups at 12- or 12-month follow-up. CAIS showed significantly higher improvement in the intervention group at 12- or 24-month follow-up in the child reports (Cohen *d* = 0.24 and 0.26, respectively) and at 12 months in the parent reports (Cohen *d* = 0.35). The between-group differences in changes in parent mental health problems were not significant. The results of the same analysis of covariance using imputed data did not differ from those shown in Table 2, excluding parent-reported social phobia at 24 months, in which no significant change was detected (eTable 7 in Supplement 1).

eTable 8 in Supplement 1 details the outcomes in the intervention and control groups when using the trial end of 6-month follow-up as the reference. In the intervention group, there was significant improvement from 6 to 12 months in SCARED-C (mean [SE] change, 3.3 [0.7]; *P* < .001) and SCARED-P (mean [SE] change, 3.8 [0.6]; *P* < .001) total scores, CAIS-C (mean [SE] change, 2.0 [0.8]; *P* = .009) and CAIS-P (mean [SE] change, 1.9 [0.5]; *P* < .001) as well as SCARED-C panic disorder subscale (mean [SE] change, 0.6 [0.3]; *P* = .01), general anxiety (mean [SE] change, 0.7 [0.2]; *P* = .004), separation anxiety (mean [SE] change, 1.0 [0.2]; *P* < .001) and social anxiety (mean [SE] change, 0.9 [0.2]; *P* < .001) and SCARED-P panic disorder subscale (mean [SE] change, 0.7 [0.2]; *P* < .001), general anxiety (mean [SE] change, 0.8 [0.2]; *P* < .001), separation anxiety (mean [SE] change, 1.2 [0.2]; *P* < .001), social anxiety (mean [SE] change, 0.8 [0.2]; *P* < .001) and school phobia (mean [SE] change, 0.3 [0.1]; *P* = .008). In the control group, the only significant changes were seen in child-reported (mean change [SE], 0.9 [0.2]; *P* < .001) and parent-reported (mean [SE] change, 0.5 [0.2]; *P* < .001) separation anxiety and parental DASS-21 total (mean [SE] change, 1.8 [0.7]; *P* = .02) and stress (mean [SE] change, 1.0 [0.4]; *P* = .005) scores.

No significant changes were observed from 6 to 24 months in the control group for the primary outcomes because only child- and parent-reported separation anxiety, parent-reported panic and general anxiety, as well as parental stress decreased.

Table 3 presents between-group comparisons of changes in primary and secondary outcomes between 6 and 12 months and 6 and 24 months. At 12 months, the mean change in the SCARED-C total score was 2.3 (95% CI, 0.3-4.2) points higher in the intervention group than in the control group (*P* = .02; Cohen *d* = 0.21), and the mean change in the SCARED-P total score was 3.3 (95% CI, 1.7-4.9) points higher (*P* < .001; Cohen *d* = 0.37). The between-group comparisons of changes in SCARED-C and SCARED-P total scores between 6 and 24 months did not show any statistically significant findings.

**Table 3.** Baseline Adjusted Comparisons of the Primary and Secondary Outcomes for the Intervention and Control Groups^a^

Outcome	Intervention vs control					
	6-12 mo Follow-up			6-24 mo Follow-up		
	Mean (95% CI)^b^	*P* value	Cohen *d*	Mean (95% CI)^b^	*P* value	Cohen *d*
**Primary**						
SCARED-C total score	2.3 (0.3 to 4.2)	.02	0.21	2.2 (−0.5 to 4.9)	.11	0.15
SCARED-P total score	3.3 (1.7 to 4.9)	<.001	0.37	2.1 (−0.1 to 4.4)	.06	0.17
**Secondary: child reported **						
SCARED-C subscores						
Panic disorder	0.9 (0.2 to 1.6)	.01	0.24	1.3 (0.3 to 2.3)	.01	0.23
General anxiety	0.4 (−0.3 to 1.0)	.28	0.10	0.5 (−0.4 to 1.4)	.29	0.10
Separation anxiety	0.2 (−0.3 to 0.7)	.46	0.07	−0.1 (−0.7 to 0.5)	.80	−0.02
Social anxiety	0.7 (0.1 to 1.3)	.01	0.23	0.5 (−0.2 to 1.2)	.19	0.12
School phobia	0.2 (−0.1 to 0.4)	.30	0.10	0.03 (−0.3 to 0.4)	.87	0.02
CDI total score	0.01 (−1.2 to 1.2)	.99	0.00	0.2 (−1.6 to 2.1)	.79	0.02
CAIS total score	0.7 (−1.4 to 2.9)	.50	0.06	1.0 (−1.7 to 3.7)	.47	0.07
**Secondary: parent reported **						
SCARED-P subscores						
Panic disorder	0.8 (0.2 to 1.4)	.01	0.24	0.2 (−0.5 to 1.0)	.58	0.05
General anxiety	0.7 (0.1 to 1.2)	.02	0.21	0.8 (0.03 to 1.6)	.04	0.19
Separation anxiety	0.6 (0.2 to 1.1)	.006	0.26	0.3 (−0.2 to 0.8)	.21	0.12
Social anxiety	0.9 (0.4 to 1.5)	<.001	0.32	0.6 (0.01 to 1.3)	.04	0.19
School phobia	0.3 (−0.003 to 0.5)	.052	0.18	0.1 (−0.3 to 0.4)	.67	0.04
CAIS total score	1.8 (0.4 to 3.2)	.01	0.24	0.4 (−1.5 to 2.4)	.67	0.04
DASS-21 scores						
Total	−1.3 (−3.4 to 0.8)	.23	−0.11	−1.6 (−4.2 to 0.9)	.21	−0.12
Depression	−0.6 (−1.5 to 0.39	.21	−0.12	−0.8 (−2.0 to 0.4)	.17	−0.13
Anxiety	−0.05 (−0.8 to 0.7)	.90	−0.01	0.3 (−0.5 to 1.1)	.52	0.06
Stress	−0.6 (−1.7 to 0.5)	.26	−0.10	−1.1 (−2.3 to 0.1)	.08	−0.16

Abbreviations: CAIS, Child Anxiety Impact Scale; CDI, Child Depression Inventory; DASS-21, Depression Anxiety and Stress Scale–21 Items; SCARED-C, Screen for Child Anxiety Related Disorders–Child version; SCARED-P, Screen for Child Anxiety Related Disorders–Parent version.

^a^ Results of the analysis of covariance.

^b^ Least-squares means.

eTable 9 in Supplement 1 presents comparisons between the intervention and control groups from the 12-month to the 24-month follow-up. No significant changes in any of the primary or secondary outcomes were observed. In the intervention group, 6-month follow-up was completed by 182 children (78%), 12-month follow-up by 170 children (73%), and 24-month follow-up by 164 children (70%), whereas 6-month follow-up in the control group was completed by 196 children (85%), 12-month follow-up by 188 (81%), and 24-month follow-up by 171 (74%). Children with suicidal intentions and families with a suspicion of child abuse, ongoing custody battle, or severe disease of a parent were excluded, composing 57 (6%) of the recruited children. At 12 months, there was a significant difference between the groups in the number of children who completed the follow-up (170 vs 188; *P* = .03), whereas no significant difference was found at 6 months (182 vs 196; *P* = .05) or at 24 months (164 vs 171; *P* = .34). We compared baseline characteristics of participants retained through 24 months and those unavailable for follow-up; these data are presented in eTable 10 in Supplement 1. Baseline characteristics were similarly associated with dropout at 24 months in the intervention and control groups.

## Discussion

The uniqueness of the current study lies in population-based screening, large sample size, guided internet-based intervention, and extended follow-up period. The study findings suggest greater effectiveness of the guided iCBT intervention compared with the psychoeducation control in both child- and parent-reported anxiety symptoms at 12 and 24 months compared with the baseline, as well as in the posttrial phase from 6 to 12 months. The between-group differences at 12 months remained the same until the 24-month follow-up.

To our knowledge, no previous RCT on iCBT for childhood anxiety has reported outcomes extending to a 24-month follow-up. The findings of the current study are in line with the 2 previous studies^10,11^ reporting 12-month follow-up data. However, the previous studies^10,11,12^ did not include a comparison between the intervention group and the control group because participants in the control group either crossed over to iCBT after the primary end point^10,11^ or their data had not been gathered after treatment.^12^ These previous studies^10,11,12^ had substantially smaller sample sizes and higher attrition rates compared with the current study.

Child reports of anxiety showed effect sizes of 0.35 and 0.29 at 12 and 24 months, respectively, compared with baseline and of 0.21 at 12 months, respectively compared with posttrial 6-month follow-up. Parent reports of child's anxiety had effect sizes of 0.36 and 0.19 at 12 and 24 months and 0.37 at 12 months when compared with posttrial 6-month follow-up. In the report on the RCT phase, the effect size at 6 months was 0.17 for child-reported anxiety, whereas differences in parent reports of anxiety were not significant.^9^ For secondary outcomes, when compared with baseline, significant changes were found on the CAIS-C and most SCARED-C subscales at 12 and 24 months. In the CAIS-P and SCARED-P subscales, all subscale scores were significant at 12 months, whereas at 24 months, only general, separation, and social anxiety subscale scores remained significantly improved. After the RCT phase, significant changes were seen in child-reported panic disorder and social anxiety symptoms from 6 to 12 months and only panic disorder symptoms from 6 to 24 months. Similarly, significant changes were seen in almost all parent-reported anxiety subscale scores and CAIS-P score at 12 months and in general and social anxiety subscale scores only at 24 months.

There are several possible explanations for the different patterns of improvement during the follow-up period. First, the previously reported improvements in parent and child evaluations in both the intervention and control groups at 6 months were significant, but the between-group differences were rather low. This finding indicates that early identification of childhood anxiety, screening, assessment, and psychoeducation already serve as an effective intervention. Second, although improvement was maintained in the psychoeducational control group at 12 and 24 months, the improvements were clearly higher in the intervention group. The substantially higher treatment gains at later follow-ups may indicate that the intervention induced a slow restructuring of the child’s and the parent’s thought processes and behavior, which became most visible at the 12-month follow-up and remained significant up to the 24-month follow-up. This delayed effectiveness may be interpreted as the psychosocial intervention producing greater improvements at later follow-ups than immediately after treatment.^24^ Additional explanations might be differences in dropout and differential changes in participant behavior, such as service use. The percentages showing the participants’ use of psychoactive medication or psychosocial support were higher in the control group than in the intervention group, but these differences were not statistically significant during the follow-up. When the dropout percentages were compared, there was a difference at 12 months between the study groups, but no differences were found at 6- or 24-month follow-ups. Baseline characteristics associated with dropout did not differ between the 2 groups. Thus, it seems unlikely that the transient difference in response rates between the intervention and control groups at 12-month follow-up would be associated with the overall results. Of note, access to the digital platform was available for both groups until the 24-month follow-up, which may have been associated with the delayed response.

The delayed clinical response to iCBT found in the current study may correlate with delayed brain alterations. Anxiety disorders are associated with changes in brain structure, such as altered volume in brain areas associated with anxiety reactivity,^25^ altered neural circuitry,^26^ and maladaptive selective information processing.^27^ Conversely, CBT has been shown to change brain function, including increased activation in the ventrolateral prefrontal cortex^28^ and regulation of the prefrontal and limbic regions to enhance emotional processing of anxiety.^29^ The neural alterations induced by CBT appear to have long-lasting potential, even continuing after the intervention has ended. It has been suggested that normalization of emotion regulation circuitry could contribute to the long-term outcomes of CBT.^30^

In this study, the iCBT intervention was associated with modest effectiveness with small effect sizes compared with digitally delivered psychoeducation. In the RCT phase of this study, those children who fulfilled the criteria of at least 1 anxiety disorder were more likely to finish all themes of the intervention and benefitted more from it than children without an anxiety disorder.^9,15^ These findings indicate that the intervention most likely benefits those children who meet the diagnostic criteria of an anxiety disorder. However, participants do not need to be specialized health care patients. In the future, children could be referred to this program from primary health care practitioners. The screening process in primary health care would make it possible to reach children and families who are not motivated or have no possibility to visit hospital clinics.

### Limitations

This study has some limitations. First, 130 participants (28%) dropped out or were unavailable for follow-up. In the sensitivity analyses, the main results remained the same when considering the implications of missing data, and the baseline characteristics associated with dropout did not differ between the 2 groups. Second, the follow-up assessments were based on parent and child reports, whereas diagnostic assessments at follow-ups for both groups would have strengthened the findings. Third, the control group received more services than is typical for preschool children with anxiety problems. Fourth, children with suicidal intentions and families with a suspicion of child abuse, ongoing custody battle, or severe disease of a parent were excluded, composing 57 (6%) of the recruited children. It is possible that some children who were excluded because of these reasons had severe anxiety. Fifth, the effect sizes were overall small. When a sample size is large, even modest changes with small effect sizes may reach statistical significance.

## Conclusions

In this cohort study of children with anxiety, the findings suggest that early identification of childhood anxiety combined with iCBT or even digital psychoeducation was associated with significant long-term improvement in children’s symptoms and daily functioning up to 24 months. At 12 months, there were significant differences in the child- and parent-reported anxiety levels of children between the iCBT and control groups, and the differences were maintained until 24 months. The findings of this study are both timely and relevant in the light of the recent increase in youth anxiety worldwide. Unlike RCTs conducted with referred populations or convenience samples, this study evaluated an early iCBT intervention based on population-based screening for children with anxiety, whose problems often go unnoticed by parents or other adults. These findings are important for both clinical practice and future intervention research, which often lacks long-term follow-up data.

## Notes

Supplement 1. **eTable 1.** Demographic characteristics of the intervention and education control groups **eTable 2.** Inclusion and exclusion criteria of the study **eTable 3.** Content of the Master Your Worries intervention **eTable 4.** Content of the shortened 5-item SCARED **eTable 5.** Mean scores of the primary and secondary outcomes at baseline, 12, and 24 months after randomization by randomized treatment group **eTable 6.** Treatment comparisons of the primary and secondary outcomes between Master Your Worries intervention and education control (EC) according to ANOVA **eTable 7.** Treatment comparisons of the primary and secondary outcomes at 12 and 24 months between Master Your Worries intervention and education control (EC) using imputed data and baseline measurement of each outcome as a covariate **eTable 8.** Mean (SE) changes in the primary and secondary outcomes from 6 months to 12 and 24 months after randomization in Master Your Worries intervention and education control (EC) group **eTable 9.** Treatment comparisons of the primary and secondary outcomes between Master Your Worries intervention and education control (EC) 12 months to 24 months adjusting for baseline measurement of each outcome as a covariate **eTable 10.** Treatment group comparisons of demographic characteristics between those with baseline and 24 months’ measurement and those with baseline without 24 months’ measurement

Supplement 2.
Data Sharing Statement
