# Supplementary material for: Internet-Based Cognitive Behavioral Therapy and Telephone Coaching for Anxiety in Children
Source: JAMA Netw Open. 2026 Aug 14;9(8):e2629195. doi: 10.1001/jamanetworkopen.2026.29195 (PMC13476841; doi:10.1001/jamanetworkopen.2026.29195)
Supplement: Supplementary file 1 [file jamanetwopen-e2629195-s001.pdf]

Supplemental Online Content

Sourander A, Kaajalaakso K, Korpilahti-Leino T, et al. Internet-based cognitive behavioral therapy and telephone coaching for anxiety in children. *JAMA Netw Open*. 2026;9(8):e2629195. doi:10.1001/jamanetworkopen.2026.29195

eTable 1. Demographic characteristics of the intervention and education control groups

eTable 2. Inclusion and exclusion criteria of the study

eTable 3. Content of the Master Your Worries intervention

eTable 4. Content of the shortened 5-item SCARED

eTable 5. Mean scores of the primary and secondary outcomes at baseline, 12, and 24 months after randomization by randomized treatment group

eTable 6. Treatment comparisons of the primary and secondary outcomes between Master Your Worries intervention and education control (EC) according to ANOVA

eTable 7. Treatment comparisons of the primary and secondary outcomes at 12 and 24 months between Master Your Worries intervention and education control (EC) using imputed data and baseline measurement of each outcome as a covariate

eTable 8. Mean (SE) changes in the primary and secondary outcomes from 6 months to 12 and 24 months after randomization in Master Your Worries intervention and education control (EC) group

eTable 9. Treatment comparisons of the primary and secondary outcomes between Master Your Worries intervention and education control (EC) 12 months to 24 months adjusting for baseline measurement of each outcome as a covariate

eTable 10. Treatment group comparisons of demographic characteristics between those with baseline and 24 months' measurement and those with baseline without 24 months' measurement

This supplemental material has been provided by the authors to give readers additional information about their work.

© 2026 Sourander A et al. *JAMA Network Open*.

eTable 1. Demographic characteristics of the intervention and education control groups

**Baseline variable**		**Intervention (n = 234)**	**Education control (n = 231)**	**p**
Child characteristics				
Sex, n (%)	Girls	167 (71.4)	165 (71.4)	
	Boys	67 (28.6)	66 (28.6)	.93
Age (years), mean (SD)		11.5 (1.0)	11.4 (0.9)	.40
Parent/Family characteristics				
Respondent, n (%)	Biological mother	212 (90.6)	209 (90.5)	
	Biological father	20 (8.5)	19 (8.2)	
	Other[1]	2 (0.9)	3 (1.3)	.96
Age (years), mean (SD)	Mother	41.9 (5.2)	42.1 (5.1)	.68
	Father	43.8 (6.7)	44.6 (6.4)	.27
Family structure (n, %)	Two biological parents	145 (62.0)	148 (64.1)	
	Non-nuclear family	89 (38.0)	83 (35.9)	.64
	No siblings	15 (6.4)	17 (7.4)	
	1 sibling or more	219 (93.6)	214 (92.6)	.69
Native language, n (%)	Finnish	228 (97.4)	217 (93.9)	
	Swedish	4 (1.7)	9 (3.9)	
	Other[2]	2 (0.9)	5 (2.2)	.21
Parental education, n (%)	Secondary education or lower	85 (36.3)	79 (34.2)	
	Higher education level	149 (63.7)	152 (65.8)	.63

[1]E.g. stepparent, foster parent, [2]The most common other native language in the sample was Estonian (2 speakers). Speakers of Greek, Hungarian, Italian, Russian, and Turkish were also represented in the sample.

© 2026 Sourander A et al. *JAMA Network Open*.

eTable 2. Inclusion and exclusion criteria of the study

**Inclusion criteria**	**Exclusion criteria**
Age 10–13	Disabling visual or hearing impairment
Daily access to internet	Intellectual disability
Fluency in Finnish or Swedish	Autism spectrum disorder
A minimum score of 22 points on the 41-item SCARED	Suicidal intentions or severe mental health disorder
	Ongoing psychotherapy or therapy due to start within 6 months
	Changes in anxiety medication within two months
	Ongoing custody battle or child abuse investigations
	Severe mental or somatic disease of a parent

SCARED = Screen for Child Anxiety Related Emotional Disorders.

© 2026 Sourander A et al. *JAMA Network Open*.

eTable 3. Content of the Master Your Worries intervention

Theme	Content
Introduction	Intervention and tools, motivation, creating working alliance, psychoeducation
Theme 1. Learn to know anxiety	Psychoeducation about anxiety and fears, CBT model
Theme 2. Deep breathing	Breathing techniques, introducing exposure, managing anxiety, gradual exposure
Theme 3. Encouraging thinking	Recognize and change negative thoughts, cognitive restructuring, gradual exposure
Theme 4. Relaxation	Relaxation techniques, managing anxiety, gradual exposure
Theme 5. Safe place	Imaginary techniques, managing anxiety, gradual exposure
Theme 6. Anxiety Ladders	Create exposure hierarchy
Theme 7. Learn by practicing	Practice in real life
Theme 8. Master your anxiety	Summarize skills, gradual exposure
Theme 9. Future plan	Create a long-term plan, prevent setbacks
Booster phone call	Reinforce skills

Note: CBT = cognitive behavioral therapy

© 2026 Sourander A et al. *JAMA Network Open*.

eTable 4. Content of the shortened 5-item SCARED

	Not true or hardly ever true (0)	Somewhat true or sometimes true (1)	Very true or often true (2)
I am nervous.[1]			
I get scared if I sleep away from home.[2]			
When I get frightened, I feel dizzy.[3]			
I am scared to go to school.[4]			
I feel nervous when I am going to any place where there will be people that I do not know well.[5]			

A total score of three or more points, or two points on any of the five items, was used as a cut-off. The selection of items was based on a Finnish epidemiological study (Kaajalaakso et al., 2021), and the statement with highest sensitivity was chosen from each of the five SCARED subscales.

[1]Generalized anxiety subscale, [2]Separation anxiety subscale, [3]Panic disorder subscale, [4]School phobia subscale, [5]Social phobia subscale.

Reference: Kaajalaakso K, Lempinen L, Ristkari T, Huttunen J, Luntamo T, Sourander A. Psychometric properties of the screen for child anxiety related emotional disorders (SCARED) among elementary school children in Finland. Scand J Psychol. 2021;62(1):34-40. doi:10.1111/sjop.12677

© 2026 Sourander A et al. *JAMA Network Open*.

eTable 5. Mean scores of the primary and secondary outcomes at baseline, 12, and 24 months after randomization by randomized treatment group

	Intervention			Education control		
	Baseline Mean[a] (SE)	12 months Mean[a] (SE)	24 months Mean[a] (SE)	Baseline Mean[a] (SE)	12 months Mean[a] (SE)	24 months Mean[a] (SE)
**Primary outcomes**						
Child-reported SCARED total score	33.3 (0.6)	18.9 (0.9)	18.8 (1.0)	33.2 (0.7)	23.3 (0.9)	23.2 (1.0)
Parent-reported SCARED total score	23.8 (0.7)	14.3 (0.7)	15.2 (0.9)	22.7 (0.7)	17.1 (0.7)	16.9 (0.9)
**Child-reported secondary outcomes**						
SCARED subscores						
Panic disorder	7.1 (0.3)	4.3 (0.3)	4.1 (0.4)	6.9 (0.3)	4.6 (0.3)	4.8 (0.4)
General anxiety	9.3 (0.2)	5.5 (0.3)	6.1 (0.3)	8.9 (0.2)	6.3 (0.3)	7.0 (0.3)
Separation anxiety	7.0 (0.2)	2.8 (0.2)	2.0 (0.2)	7.3 (0.2)	4.1 (0.2)	3.1 (0.2)
Social anxiety	7.6 (0.2)	4.8 (0.3)	5.2 (0.3)	7.8 (0.2)	6.6 (0.3)	6.7 (0.3)
School phobia	2.3 (0.1)	1.4 (0.1)	1.4 (0.1)	2.2 (0.1)	1.7 (0.1)	1.6 (0.1)
CDI total score	12.1 (0.6)	9.2 (0.6)	9.8 (0.7)	12.1 (0.6)	10.0 (0.6)	10.9 (0.7)
CAIS total score	19.0 (0.8)	9.2 (0.8)	10.9 (0.9)	19.9 (0.8)	12.5 (0.8)	14.5 (0.9)
**Parent-reported secondary outcomes**						
SCARED subscores						
Panic disorder	3.4 (0.2)	2.1 (0.3)	2.8 (0.3)	3.1 (0.2)	2.6 (0.2)	2.7 (0.3)
General anxiety	7.6 (0.3)	5.0 (0.3)	5.1 (0.3)	7.2 (0.3)	5.5 (0.3)	5.7 (0.3)
Separation anxiety	5.0 (0.2)	2.0 (0.2)	1.6 (0.2)	4.9 (0.2)	2.8 (0.2)	2.1 (0.2)
Social anxiety	5.7 (0.3)	3.8 (0.3)	4.4 (0.3)	5.7 (0.3)	4.8 (0.3)	5.0 (0.3)
School phobia	2.1 (0.1)	1.2 (0.1)	1.3 (0.1)	1.9 (0.1)	1.3 (0.1)	1.3 (0.1)
CAIS total score	14.6 (0.6)	7.6 (0.7)	9.3 (0.8)	14.4 (0.6)	10.4 (0.6)	10.7 (0.8)
DASS						
Total	15.9 (1.0)	14.1 (1.0)	15.0 (1.1)	14.2 (1.0)	13.0 (1.0)	13.5 (1.1)
Depression	4.6 (0.4)	4.1 (0.4)	4.7 (0.5)	4.1 (0.4)	3.6 (0.4)	3.9 (0.5)
Anxiety	3.0 (0.3)	2.4 (0.3)	2.4 (0.3)	2.5 (0.3)	2.2 (0.3)	2.5 (0.3)
Stress	8.4 (0.4)	7.6 (0.5)	7.9 (0.5)	7.6 (0.4)	7.2 (0.5)	7.0 (0.5)

Note: CAIS = Child Anxiety Impact Scale; CDI = Child Depression Inventory; DASS-21 = Depression Anxiety and Stress Scale Short Form; SCARED = Screen for Child Anxiety Related Emotional Disorders. [a] Least-squares means.

© 2026 Sourander A et al. *JAMA Network Open*.

eTable 6. Treatment comparisons of the primary and secondary outcomes between Master Your Worries intervention and education control (EC) according to ANOVA

	Intervention vs. Education control					
	Baseline to 12 months			Baseline to 24 months		
	Mean[a] (95% CI)	p	Effect size	Mean[a] (95% CI)	p	Effect size
**Primary outcomes**						
Child-reported SCARED total score	**4.6 (2.2, 7.1)**	**<.001**	**0.34**	**4.5 (1.5, 7.5)**	**0.003**	**0.28**
Parent-reported SCARED total score	**3.9 (2.1, 5.8)**	**<.001**	**0.38**	**2.8 (0.3, 5.2)**	**0.026**	**0.21**
**Child-reported secondary outcomes**						
SCARED subscores						
Panic disorder	0.5 (-0.4, 1.4)	0.29	0.10	0.9 (-0.2, 2.0)	0.12	0.14
General anxiety	**1.2 (0.4, 2.0)**	**0.005**	**0.26**	**1.3 (0.3, 2.2)**	**0.012**	**0.24**
Separation anxiety	**1.0 (0.4, 1.7)**	**<.001**	**0.31**	**0.8 (0.1, 1.4)**	**0.017**	**0.22**
Social anxiety	**1.6 (0.9, 2.2)**	**<.001**	**0.42**	**1.3 (0.5, 2.1)**	**0.001**	**0.30**
School phobia	**0.4 (0.01, 0.7)**	**0.046**	**0.19**	0.3 (-0.1, 0.7)	0.20	0.12
CDI total score	0.9 (-0.7, 2.4)	0.28	0.10	1.1 (-0.9, 3.1)	0.27	0.10
CAIS total score	2.4 (-0.04, 4.9)	0.054	0.18	2.7 (-0.04, 5.5)	0.053	0.18
**Parent-reported secondary outcomes**						
SCARED subscores						
Panic disorder	**0.9 (0.2, 1.5)**	**0.008**	**0.25**	0.3 (-0.5, 1.1)	0.48	0.07
General anxiety	**0.9 (0.2, 1.6)**	**0.007**	**0.25**	**1.1 (0.3, 1.9)**	**0.008**	**0.25**
Separation anxiety	**0.9 (0.3, 1.4)**	**0.003**	**0.28**	0.6 (-0.04, 1.2)	0.067	0.17
Social anxiety	**1.0 (0.4, 1.5)**	**<.001**	**0.31**	0.7 (-0.1, 1.4)	0.070	0.17
School phobia	**0.4 (0.1, 0.7)**	**0.013**	**0.23**	0.2 (-0.2, 0.6)	0.36	0.09
CAIS total score	**3.0 (1.4, 4.7)**	**<.001**	**0.34**	1.7 (-0.5, 3.8)	0.12	0.14
DASS						
Total	0.6 (-1.7, 2.8)	0.61	0.05	0.2 (-2.4, 2.8)	0.86	0.02
Depression	-0.2 (-1.1, 0.8)	0.75	0.03	-0.4 (-1.6, 0.8)	0.52	0.06
Anxiety	0.3 (-0.4, 1.1)	0.38	0.08	0.6 (-0.2, 1.5)	0.14	0.14
Stress	0.4 (-0.8, 1.5)	0.53	0.06	-0.1 (-1.3, 1.1)	0.85	0.02

Note: CAIS = Child Anxiety Impact Scale; CDI = Child Depression Inventory; DASS-21 = Depression Anxiety and Stress Scale Short Form; SCARED = Screen for Child Anxiety Related Emotional Disorders.

Statistically significant differences are shown in bold.

[a] Least-squares means

© 2026 Sourander A et al. *JAMA Network Open*.

eTable 7. Treatment comparisons of the primary and secondary outcomes at 12 and 24 months between Master Your Worries intervention and education control (EC) using imputed data and baseline measurement of each outcome as a covariate

	Intervention vs. Education control					
	12 months			24 months		
	Mean[a] (95% CI)	p	Effect size	Mean[a] (95% CI)	p	Effect size
**Primary outcomes**						
Child-reported SCARED total score	**4.6 (2.1, 7.0)**	**<.001**	**0.33**	**4.6 (1.7, 7.5)**	**0.002**	**0.29**
Parent-reported SCARED total score	**3.6 (1.8, 5.4)**	**<.001**	**0.36**	**2.3 (0.1, 4.5)**	**0.043**	**0.18**
**Child-reported secondary outcomes**						
SCARED subscores						
Panic disorder	0.3 (-0.6, 1.2)	0.48	0.06	0.8 (-0.3, 1.8)	0.15	0.13
General anxiety	**1.0 (0.1, 1.8)**	**0.022**	**0.21**	**1.1 (0.1, 2.2)**	**0.035**	**0.21**
Separation anxiety	**1.3 (0.7, 1.8)**	**<.001**	**0.41**	**1.0 (0.5, 1.6)**	**<.001**	**0.33**
Social anxiety	**1.7 (1.1, 2.4)**	**<.001**	**0.45**	**1.4 (0.7, 2.2)**	**<.001**	**0.35**
School phobia	**0.3 (0.003, 0.7)**	**0.048**	**0.17**	0.2 (-0.1, 0.6)	0.22	0.11
CDI total score	0.9 (-0.6, 2.4)	0.25	0.10	1.4 (-0.5, 3.3)	0.15	0.13
CAIS total score	**2.7 (0.6, 4.8)**	**0.011**	**0.21**	**3.3 (0.6, 6.1)**	**0.018**	**0.23**
**Parent-reported secondary outcomes**						
SCARED subscores						
Panic disorder	**0.7 (0.05, 1.4)**	**0.036**	**0.20**	0.1 (-0.6, 0.9)	0.78	0.03
General anxiety	**0.7 (0.1, 1.3)**	**0.014**	**0.21**	**0.9 (0.2, 1.6)**	**0.013**	**0.21**
Separation anxiety	**0.8 (0.3, 1.3)**	**<.001**	**0.31**	**0.5 (0.04, 1.0)**	**0.035**	**0.19**
Social anxiety	**1.0 (0.5, 1.5)**	**<.001**	**0.32**	0.6 (-0.002, 1.2)	0.051	0.17
School phobia	**0.3 (0.02, 0.5)**	**0.033**	**0.19**	0.1 (-0.3, 0.4)	0.71	0.03
CAIS total score	**3.1 (1.7, 4.6)**	**<.001**	**0.36**	1.7 (-0.3, 3.6)	0.096	0.15
DASS						
Total	-0.04 (-2.1, 2.1)	0.97	0.00	-0.5 (-2.9, 1.9)	0.69	0.03
Depression	-0.3 (-1.2, 0.6)	0.52	0.06	-0.5 (-1.7, 0.6)	0.36	0.08
Anxiety	0.1 (-0.6, 0.7)	0.80	0.02	0.5 (-0.3, 1.2)	0.25	0.10
Stress	0.1 (-1.0, 1.1)	0.89	0.01	-0.5 (-1.6, 0.6)	0.36	0.08

Note: CAIS = Child Anxiety Impact Scale; CDI = Child Depression Inventory; DASS-21 = Depression Anxiety and Stress Scale Short Form; SCARED = Screen for Child Anxiety Related Emotional Disorders.

Statistically significant differences are shown in bold.

[a] Baseline adjusted least-squares means

© 2026 Sourander A et al. *JAMA Network Open*.

eTable 8. Mean (SE) changes in the primary and secondary outcomes from 6 months to 12 and 24 months after randomization in Master Your Worries intervention and education control (EC) group

	**Intervention**				**Education control**			
	**6 months to 12 months**		**6 months to 24 months**		**6 months to 12 months**		**6 months to 24 months**	
	**Mean[a] (SE)**	**p**	**Mean[a] (SE)**	**p**	**Mean[a] (SE)**	**p**	**Mean[a] (SE)**	**p**
**Primary outcomes**								
Child-reported SCARED total score	**3.3 (0.7)**	**<.001**	**3.3 (1.0)**	**<.001**	1.0 (0.7)	0.15	1.1 (1.0)	0.25
Parent-reported SCARED total score	**3.8 (0.6)**	**<.001**	**2.8 (0.8)**	**<.001**	0.5 (0.6)	0.35	0.8 (0.8)	0.33
**Child-reported secondary outcomes**								
SCARED subscores								
Panic disorder	**0.6 (0.3)**	**0.011**	**0.8 (0.4)**	**0.038**	-0.3 (0.2)	0.29	-0.5 (0.4)	0.14
General anxiety	**0.7 (0.2)**	**0.004**	0.1 (0.3)	0.70	0.3 (0.2)	0.15	-0.3 (0.3)	0.29
Separation anxiety	**1.0 (0.2)**	**<.001**	**1.8 (0.2)**	**<.001**	**0.9 (0.2)**	**<.001**	**1.9 (0.2)**	**<.001**
Social anxiety	**0.9 (0.2)**	**<.001**	0.5 (0.3)	0.057	0.2 (0.2)	0.44	0.0 (0.3)	0.93
School phobia	0.1 (0.1)	0.46	0.1 (0.1)	0.33	-0.1 (0.1)	0.43	0.1 (0.1)	0.60
CDI total score	-0.2 (0.4)	0.59	-0.9 (0.7)	0.18	-0.3 (0.4)	0.54	-1.2 (0.7)	0.076
CAIS total score	**2.0 (0.8)**	**0.009**	0.4 (1.0)	0.70	1.3 (0.8)	0.077	-0.6 (1.0)	0.51
**Parent-reported secondary outcomes**								
SCARED subscores								
Panic disorder	**0.7 (0.2)**	**<.001**	0.1 (0.3)	0.69	-0.04 (0.2)	0.85	-0.1 (0.3)	0.74
General anxiety	**0.8 (0.2)**	**<.001**	**0.8 (0.3)**	**0.006**	0.2 (0.2)	0.38	-0.02 (0.3)	0.95
Separation anxiety	**1.2 (0.2)**	**<.001**	**1.6 (0.2)**	**<.001**	**0.5 (0.2)**	**<.001**	**1.2 (0.2)**	**<.001**
Social anxiety	**0.8 (0.2)**	**<.001**	0.3 (0.2)	0.23	-0.1 (0.2)	0.54	-0.3 (0.2)	0.12
School phobia	**0.3 (0.1)**	**0.008**	0.1 (0.1)	0.38	-0.01 (0.1)	0.90	0.03 (0.1)	0.78
CAIS total score	**1.9 (0.5)**	**<.001**	0.2 (0.7)	0.81	0.1 (0.5)	0.86	-0.2 (0.7)	0.73
DASS								
Total	0.5 (0.8)	0.54	-0.4 (0.9)	0.68	**1.8 (0.7)**	**0.018**	1.3 (0.9)	0.17
Depression	-0.1 (0.3)	0.77	-0.6 (0.4)	0.15	0.5 (0.3)	0.13	0.2 (0.4)	0.63
Anxiety	0.1 (0.3)	0.56	0.2 (0.3)	0.58	0.2 (0.2)	0.44	-0.1 (0.3)	0.71
Stress	0.4 (0.4)	0.27	0.1 (0.4)	0.82	**1.0 (0.4)**	**0.005**	**1.2 (0.4)**	**0.006**

Note: CAIS = Child Anxiety Impact Scale; CDI = Child Depression Inventory; DASS-21 = Depression Anxiety and Stress Scale Short Form; SCARED = Screen for Child Anxiety Related Emotional Disorders. [a] Least-squares mean

© 2026 Sourander A et al. *JAMA Network Open*.

eTable 9. Treatment comparisons of the primary and secondary outcomes between Master Your Worries intervention and education control (EC) 12 months to 24 months adjusting for baseline measurement of each outcome as a covariate

	Intervention vs. Education control	
	12 months to 24 months	
	Mean[a] (95% CI)	P
**Primary outcomes**		
Child-reported SCARED total score	-0.1 (-2.6, 2.5)	0.95
Parent-reported SCARED total score	-1.1 (-3.1, 0.8)	0.25
**Child-reported secondary outcomes**		
SCARED subscores		
Panic disorder	0.4 (-0.5, 1.4)	0.39
General anxiety	0.1 (-0.7, 0.9)	0.78
Separation anxiety	-0.3 (-0.8, 0.3)	0.34
Social anxiety	-0.2 (-0.9, 0.5)	0.54
School phobia	-0.1 (-0.5, 0.2)	0.51
CDI total score	0.2 (-1.4, 1.9)	0.78
CAIS total score	0.3 (-2.2, 2.7)	0.83
**Parent-reported secondary outcomes**		
SCARED subscores	-0.6 (-1.2, 0.1)	0.12
Panic disorder	0.2 (-0.5, 0.8)	0.63
General anxiety	-0.3 (-0.7, 0.1)	0.17
Separation anxiety	-0.3 (-0.9, 0.3)	0.31
Social anxiety	-0.2 (-0.5, 0.1)	0.27
School phobia		
CAIS total score	-1.4 (-3.1, 0.3)	0.12
DASS		
Total	-0.4 (-2.5, 1.8)	0.75
Depression	-0.2 (-1.1, 0.7)	0.63
Anxiety	0.3 (-0.4, 1.1)	0.40
Stress	-0.5 (-1.7, 0.7)	0.43

Note: CAIS = Child Anxiety Impact Scale; CDI = Child Depression Inventory; DASS-21 = Depression Anxiety and Stress Scale Short Form; KINDL = 24-item Revised Children Quality of Life Questionnaire; SCARED = Screen for Child Anxiety Related Emotional Disorders; SDQ = Strengths and Difficulties Questionnaire.
[a] Baseline adjusted least-squares means

© 2026 Sourander A et al. *JAMA Network Open*.

eTable 10. Treatment group comparisons of demographic characteristics between those with baseline and 24 months' measurement and those with baseline without 24 months' measurement

	Baseline but not 24 months' measurement		Baseline and 24 months' measurement			
	**Intervention (n=70)**	**Education control (n=60)**	**Intervention (n=164)**	**Education control (n=171)**	**Main effect p-value**	**Interaction[a] p-value**
**Child characteristics**						
Sex, n (%)						
Boy	19 (27.1)	18 (30.0)	48 (29.3)	48 (28.1)	0.78	0.66
Girl	51 (72.9)	42 (70.0)	116 (70.7)	123 (71.9)		
Age, mean (SD)	11.5 (1.0)	11.4 (0.9)	11.5 (1.0)	11.4 (0.9)	0.94	0.84
DAWBA, n (%)						
No anxiety disorder	18 (26.1)	11 (18.6)	34 (21.1)	39 (22.9)	0.49	0.29
Any anxiety disorder	51 (73.9)	48 (81.4)	127 (78.9)	131 (77.1)		
**Parent/family characteristics**						
Age, mean (SD)						
Mother	41.4 (5.9)	42.1 (5.4)	42.1 (4.9)	42.1 (5.1)	0.51	0.49
Father	43.1 (7.3)	44.2 (4.8)	44.1 (6.5)	44.7 (6.8)	0.38	0.71
Family structure, n (%)						
Two biological parents	39 (55.7)	32 (53.3)	106 (64.6)	116 (67.8)	0.045	0.57
Other	31 (44.3)	28 (46.7)	58 (35.4)	55 (32.2)		
Respondent's education, n (%)						
Secondary or lower	31 (44.3)	25 (41.7)	54 (32.9)	54 (31.6)	0.16	0.92
Higher education	39 (55.7)	35 (58.3)	110 (67.1)	117 (68.4)		

[a] Testing treatment group-by-availability of 24 months' measurement interaction effect.

© 2026 Sourander A et al. *JAMA Network Open*.
