# Supplementary material for: Internet-Based Cognitive Behavioral Therapy and Telephone Coaching for Anxiety in Children
Source: JAMA Netw Open. 2026 Aug 14;9(8):e2629195. doi: 10.1001/jamanetworkopen.2026.29195 (PMC13476841; doi:10.1001/jamanetworkopen.2026.29195)
Supplement: Supplementary file 2 [file jamanetwopen-e2629195-s002.pdf]

Data Sharing Statement

Sourander. Internet-Based Cognitive Behavioral Therapy and Telephone Coaching for Anxiety in Children. *JAMA Netw Open*. Published August 14, 2026.
doi:10.1001/jamanetworkopen.2026.29195

Data
Data available: No

Additional Information
Explanation for why data not available: The datasets generated or analyzed during this study are not publicly available due to study privacy policy and data protection regulations (including GDPR), but summary measures are available from the corresponding author on reasonable request.
